# Adult laryngeal Embryonal Rhabdomyosarcoma: a case report and literature review

**DOI:** 10.1186/s12893-020-00831-7

**Published:** 2020-07-31

**Authors:** Juanjuan Hu, Dan Lu, Jia Ren, Qiao Wen, Jing Zhou, Weigang Gan, Jun Liu, Shixi Liu, Hui Yang, Jian Zou

**Affiliations:** Department of Otolaryngology, Head and Neck Surgery, West China Hospital, West China Medical School, Sichuan University, No. 37 Guo Xue Xiang, Chengdu, Sichuan 610041 PR China

**Keywords:** Rhabdomyosarcoma, Laryngeal rhabdomyosarcomas, Multidiscipline, Anticancer drug sensitivity

## Abstract

**Background:**

Laryngeal rhabdomyosarcomas (RMSs) mainly occurred in children, while were extremely rare in adults. Consequently, less information was available to guide clinicians to manage adult RMSs in larynx.

**Case presentation:**

A 42-year-old man presented with a 2-year history of gradually worsening hoarseness. Then, he underwent a surgery with suspension laryngoscope with initially being diagnosed as vocal cord cyst. Unexpectedly, the lesion was proved to be embryonal rhabdomyosarcoma (ERMS), pathologically. Next, he underwent chemoradiotherapy, while the tumor relapsed 18 months after the last treatment. Subsequently, a vertical hemilaryngectomy and a right selective neck dissection was performed, and the chemotherapy according to the anticancer drug sensitivity in vitro was arranged. Until the last check-up 18 months after chemotherapy, the patient did not display clinical or radiological signs of local recurrence and metastases.

**Conclusions:**

Misdiagnosis and missed diagnosis of laryngeal RMSs might appear when tumors presented as smooth protuberance. We reported the first case of laryngeal RMSs in an adult with the multidisciplinary strategy based on the chemosensitivity assay in vitro. Furthermore, a systematic review of the literature was also discussed, highlighting the initial diagnostic pitfalls and subsequent management problems that may occur with this uncommon tumor.

## Background

Primary laryngeal sarcomas constitute less than 1% of all malignant laryngeal tumors [1], and more than 50% of these tumors are fibrosarcomas, followed by osteosarcomas, chondrosarcomas, liposarcomas, leiomyosarcomas, and rhabdomyosarcomas (RMSs) [2]. RMSs usually involve in skeletal muscle, and thus can be localized in almost any site [3]. Around 40% cases originate in the head and neck region, while RMSs arising in the larynx are extremely rare [4, 5]. Laryngeal RMSs, arising from undifferentiated mesodermal tissue, mainly occurred in children. To date, only 22 cases of laryngeal RMSs in adults have been reported **(**Table 1**)**. As a result, less information was available to guide clinicians to manage adult RMSs in larynx. We reported the first case of laryngeal RMSs in an adult with the multidisciplinary strategy based on the chemosensitivity assay in vitro.

**Table 1:** Reported cases of laryngeal rhabdomyosarcoma in adults

Author/YR	Age/sex	Primary Location	Symptoms or signs	Histologic subtype	Treatment	Status by end of follow up
Mungan S [1], 2016	64/M	Glottic	Hoarseness for 2 months	Pleomorphic	Total laryngectomy with left radical neck dissection and radiotherapy (6MV photons in an Elekta Synergy Platform linear accelerator)	Alive & disease-free, 24 months
Chiramel GK [6], 2015	70/M	Supraglottic	Hoarseness for 2 years	Pleomorphic	Chemotherapy (protocol was not provided)	Loss to follow-up
Li Y [7], 2015	22/F	Glottic	Hoarseness for 1 month	Embryonal	Right cordectomy and selective neck dissection	Alive & disease-free, 60 months
Russell JO [8], 2015	45/M	Glottic	Hoarseness for 11 months	Embryonal	Chemoradiation (vincristine, actinomycin, and cyclophosphamide combined with radiation dose of 50.4 Gy)	Alive, 34 months
Kukwa W [4], 2011	33/M	Supraglottic	Longstanding hoarseness	Embryonal	Hemilaryngectomy and chemotherapy	Alive & disease-free, 62 months
Leventhal DD [9], 2010	54/F	Glottic	Hoarseness for 9 months	Alveolar	Microsurgery by CO2 laser	Alive & disease-free, 12 months
Pittore B [3], 2010	75/M	Glottic	Dysphonia for 1 year	Pleomorphic	Partial left laryngectomy	Alive & disease-free, 9 months
Papacharalampous GX [10], 2009	83/F	Subglottic	Progressive dyspnoea for 3 months	Pleomorphic	Emergency tracheostomy and endoscopic tumour excision	dead (disease-free), 16 months
Schrock A [2], 2007	60/M	Glottic	Hoarseness for 1 months	Pleomorphic	Total laryngectomy with a bilateral selective neck dissection	Alive & disease-free, 20 months
	66/M	Glottic	Dyspnea for 6 weeks and hoarseness for 2 weeks	Pleomorphic	Microsurgery by CO2 laser	Alive & disease-free, 20 months
Dikbas O [11], 2005	28/M	Glottic	Hoarseness for 15 days	Embryonal	Tracheostomy with biopcy and chemoradiation (cisplatin, vincristine, doxorubicin, and Cyclophosphamide; protocol of radiotherapy was not provided)	Alive & disease-free, 22 months
Libera DD [12], 1999	66/M	Supraglottic	Hoarseness and sense of throat fullness	Embryonal (botryoid)	Partial laryngectomy	Alive & disease-free, 10 years
Ruske DR [13], 1998	66/F	Supraglottic	Hoarseness and dyspnoea for 2 months	Pleomorphic	Total laryngectomy and radiotherapy (7040 cGys)	Alive & disease-free, 30 months
Akyol MU [14], 1998	63/M	Supraglottic	Hoarseness and increasing dyspnea for 3 months	Pleomorphic	Total laryngectomy with right modified radical neck dissection and radiotherapy (6000 cGy to the tumor bed and 5000 cGy to the neck was given with cobalt 60 at a dose of 200 cGy/day)	Died of extensive lung metastases, 8 months
Da Mosto MC [15], 1996	69/M	Glottic	Progrossive dysphonia for 7 months	Pleomorphic	Total laryngectomy and radiotherapy (6000 cGy over 6 weeks in 30 fractions)	Alive & disease-free, 24 months
Balázs M [16], 1989	40/M	Supraglottic	Hoarseness several years	Embryoid (Botryoid)	Microsurgery	Alive & disease-free, 36 months
Haerr RW [17], 1987	62/M	Supraglottic	Dysphagia several days	Alveolar	Total laryngectomy with left radical neck dissection and radiotherapy ((4000 cGy in 22 fractions)	Died of extensive distant metastases, 5 months
Srinivasan U [18], 1979	55/M	Supraglottic	Hoarseness, dysphagia and a burning sensation in the throat for two months	Pleomorphic	Laryngectomy planned	Died prior to surgery of acute laryngeal obstruction
Winter LK [19], 1978	72/M	Glottic	Hoarseness 2 months	Pleomorphic	Radical removal	Alive & disease-free, 9 months
Frugoni (Verona) [20] P, 1976	33/M	Supraglottic	Hoarseness 3 months	Pleomorphic	Partial laryngectomy and radiotherapy (protocol was not provided)	Alive & disease-free, 6 years
Hall-Jones J [21], 1975	54/M	Supraglottic	dramatically with a large tumour mass obstructing his laryngeal inlet	Embryonal	Total laryngectomy	Alive & disease-free, 15 months
Rodriguez LA [22], 1970	57/M	Glottic	Acute respiratory distress	Pleomorphic	Total laryngectomy	No follow-up reported

*Abbreviations*: *YR* Year, *F* Female, *M* Male, *Gy* Gray, *cGy* Centigray

## Case presentation

**Fig. 1:**
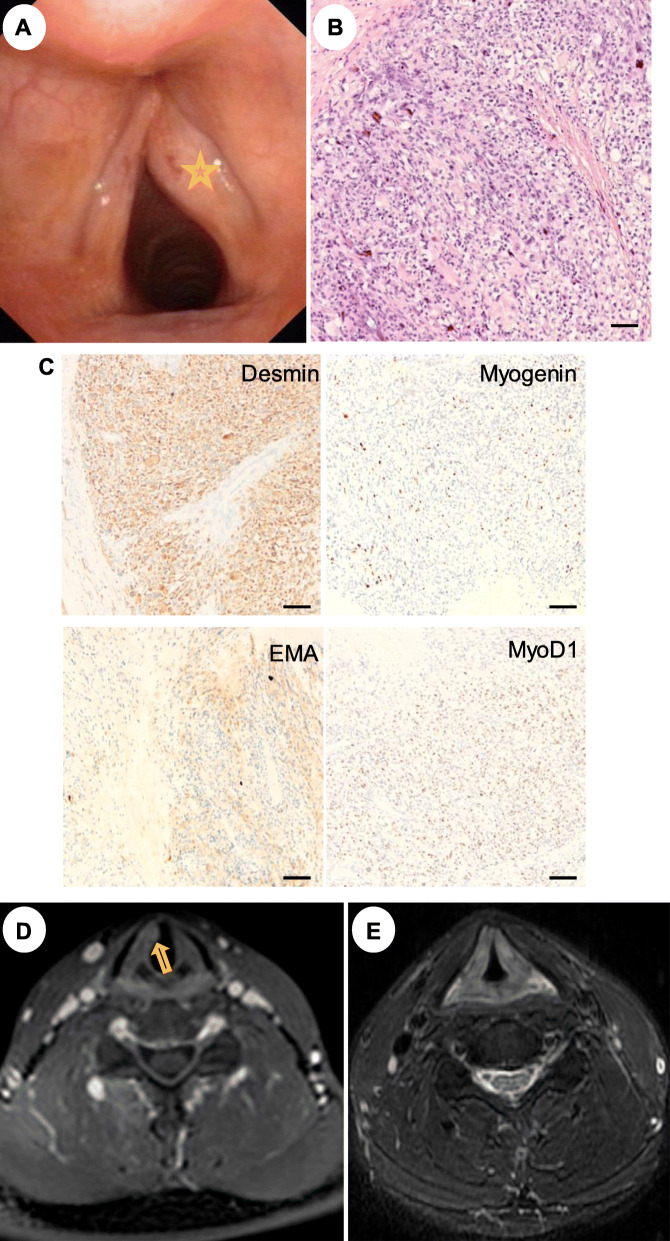
Imaging of initial treatment of this new reported case. **a**, The lesion (labeled with asterisk) was covered with normal mucosa without any ulcerations by flexible laryngoscopy. **b**, The majority of the tumor consisted of spindle-shaped cells with nucleus located centrally and eosinophilic-abundant cytoplasm (HE staining × 20). **c**, Immunohistochemistry was positive for myogenin, EMA, desmin and myoD1 (Original magnification for all slides is ×20.) **d**, MRI imaging showed the right vocal cord swelling several days after the first operation. **e**, On evaluations with MRI 1 year after the first multi-treatment, there was complete regression of the disease. Scale: 50um

A 42-year-old man presented with a 2-year history of gradually worsening hoarseness without pain, dyspnoea or dysphagia. He had no history of alcohol or tobacco use. Flexible laryngoscopy in January 2016 showed a reddish, smooth and submucosal mass with protrusion medially in the anterior third of the right vocal cord, and glottic closure was incomplete posteriorly with normal mobility (Fig. 1a). Given the well-circumscribed and relatively benign appearance, the lesion was initially suspected to be vocal cord cyst. Then the patient was planned to undergo surgery with suspension laryngoscope. Unexpectedly, the tumor was found to be fleshy, crisp and easily bleeding without invading anterior commissure. Therefore, a frozen pathology was sent during the operation, and indicated malignancy (Fig. 1b). However, the definitive pathologic diagnosis could not be made in the operation. Besides, the scope of the lesion and the lymph nodes of the neck was not defined, due for the lack of preoperative computed tomography (CT) / the magnetic resonance imaging (MRI). Therefore, the combined therapy regimen (surgery, chemotherapy or radiotherapy) cannot be formulated. Ultimately, we performed a cordectomy with the visible lesion for the patient. The final diagnose was embryonal rhabdomyosarcoma (ERMS) with immunohistochemical staining positivity for myogenin, smooth muscle actin (EMA), desmin and myoD1 (Fig. 1c). In order to develop further therapeutic regime, the MRI was performed, and showed an asymmetric swelling of the right vocal cord with no lymphadenopathy being detected in the neck (Fig. 1d). After evaluation of the tumor size, grade, and lymph node involvement, we recommended extended resection or combined chemoradiotherapy, and the patient chose the latter. He underwent three cycles of induction chemotherapy with TDF regimen (paclitaxel, cisplatin and fluorouracil) at 3-week intervals. In addition, he received a mean radiation dose of 70 Gray (Gy) to the laryngeal area. After treatment, the larynx presented with smooth appearance and good mobility of vocal cord.

**Fig. 2:**
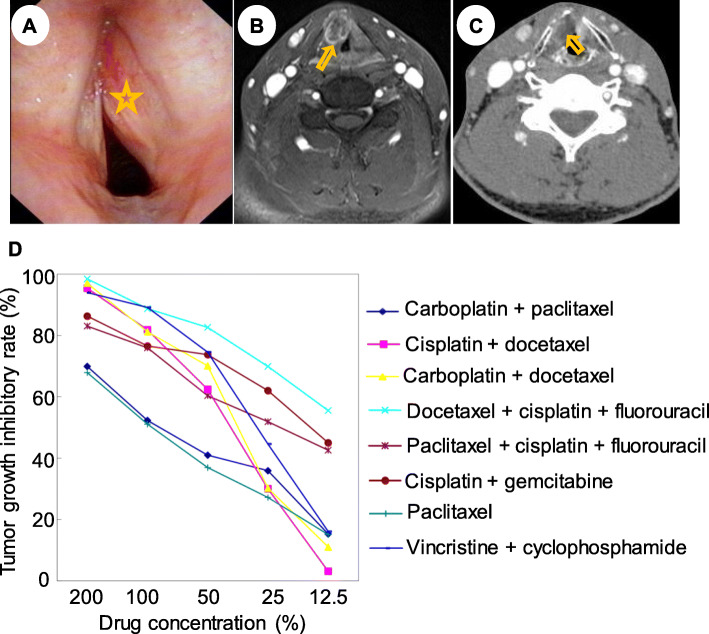
Imaging of second treatment of this new reported case. **a**, Flexible laryngoscopy revealed that a submucosal mass bulged the right true vocal cord medially and pressed the left true vocal fold with no restrictive mobility of bilateral vocal cords. **b**, MRI imaging showed that the lesion caused a partial obstruction at the level of the glottis with the size of 1.6 × 1.2 × 1.7 cm. **c**, No evidence of thyroid cartilage invasion was detected in the CT imaging. **d**, Anticancer drug sensitivity of this tumor to eight chemotherapeutic regimens was conducted, and revealed that docetaxel, cisplatin and fluorouracil was the most efficient

**Fig. 3:**
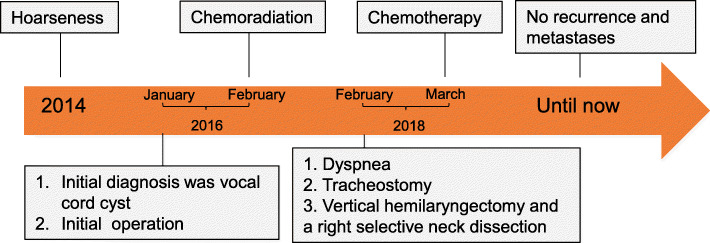
The timeline of medical record of the new reported case

The patient was followed up as scheduled with no recurrence (Fig. 1e). One year later, he lost follow-up until the presence of dyspnea 18 months after the last treatment. Flexible laryngoscopy (Fig. 2a), MRI imaging (Fig. 2b) and CT imaging (Fig. 2c) showed a submucosal mass of the right vocal cord. Intraoperative findings showed that the tan mass originated from the right vocal cord, invaded the anterior commissure and bulged into right perilaryngeal soft tissues without involving the laryngeal cartilage. Therefore, a vertical hemilaryngectomy and a right selective neck dissection was performed. No tumor cells were detected along the surgical margin and in the lymph nodes of the neck. Histological examination of the whole specimen postoperatively confirmed the diagnosis of ERMS. The tumor was clinically staged as T_3_N_0_M_0_ (stage III) glottic ERMS. Then the experiment of anticancer drug sensitivity of this tumor to eight chemotherapeutic regimens was determined in vitro, revealing that DCF regimen (docetaxel, cisplatin and fluorouracil) was the most efficient (Fig. 2d). Finally, the patient underwent DCF regimen at 3-week intervals without radiotherapy. Until the last check-up 18 months after chemotherapy, the patient did not display clinical or radiological signs of local recurrence and metastases (Fig. 3).

## Discussion and conclusions

The first case of laryngeal RMS was reported by Glick in 1944 [22]. Subsequently, most of these reported cases have occurred in children and more than 70% of RMSs were diagnosed younger than 10 years. In contrast, laryngeal RMSs occurred very infrequently in adults [13], and were more commonly found in males than females. Reviewing the 22 well-documented cases in the English-language literature, 18 cases were males (81.8%) versus 4 cases of female (18.2%). Meanwhile, laryngeal RMSs in adults were predominantly reported in the glottic and supraglottic region **(**Table 2**)**. The clinical features of the case in this study were similar with previous cases.

**Table 2:** Summary of reported cases of laryngeal rhabdomyosarcoma in adults

Characteristic	Total (NO.)	Proportion (%)
Sex		
M	18	81.81
F	4	18.18
Primary location		
Supraglottic	10	45.45
Glottic	11	50.00
Subglottic	2	4.55
Histologic subtype		
Embryonal	7	31.82
Pleomorphic	13	59.09
Alveolar	2	9.09

*Abbreviations*: *F* Female, *M* Male

The clinical features and macroscopic appearances do not differ substantially from the other laryngeal tumors, therefore, the initial diagnosis of laryngeal RMS in adults may be difficult [13]. The most common symptoms and signs, including hoarseness, dyspnea, stridor, dysphagia and polypoid appearance, provided limited diagnostic information [11]. To review the literature, RMSs had been misdiagnosed as hemangioma [23] and laryngopharyngeal reflux [7]. Our case was initially diagnosed as the vocal cord cyst, due for the cystic appearance, and the overlying mucosa was smooth. Therefore, stroboscopy or narrow-band imaging was not performed. This was the main reason of misdiagnosis, which reminded us that for vocal cord tumor, diagnosis should be combined with stroboscopy, narrow band imaging and other comprehensive evaluations, instead of clinical experience or the appearance. CT was often preferred with the information of whether laryngeal cartilage or bone erosion [10]. MRI enabled us to perform a noninvasive assessment of tumor size, localization, nerve or vascular invasion. While CT/ MRI proved to be incapable of distinguishing RMSs from other laryngeal malignant tumors.

Therefore, the definite diagnosis is based on optical microscope and immunohistochemistry [2]. Positivity for vimentin indicates the mesenchymal origin, and the staining for actin, desmin, myogenin and myoglobin suggests muscular differentiation. The histological classification of RMS is controversial. Most authors accepted the system proposed by Horn and Enterline [24] that there were three principal histological varieties of RMS: embryonal, alveolar, pleomorphic according to their degree of cellular differentiation and maturity; both spindle cell RMSs and botryoid RMSs were considered to be subtypes of embryonal RMSs [25]. The embryonal subtype is the most frequent, accounting for 70–75% of all RMSs, followed by the alveolar (20–25%) and pleomorphic differentiation (5%) [2]. To date, there were 22 cases of laryngeal RMS in adults, including 13 (59.09%) pleomorphic RMS, 7 (31.82%) embryonal RMS and 2 (9.09%) alveolar RMS **(**Table 2**)**. The case described here was an adult with the type of ERMS, which did not accord with this general pattern concerning the preponderance of age and pathological type. ERMS of the larynx must be differentiated from other common histological types in that it differs significantly with respect to its management [4].

The management of RMSs has evolved from radical surgery to less morbid regimen, which is now typically limited to organ-sparing procedures supplemented with chemoradiotherapy. This paradigm shift can be attributed to the multimodality protocols initiated by the Intergroup Rhabdomyosarcoma Study Group (IRSG) over several decades [9, 26, 27]. Since RMSs are rare in the adult population, there is no standard adult-specific chemoradiation protocols, so far, most of the chemoradiation protocols refer to pediatric series. The treatment responses of radiotherapy were variable and difficult to interpret, as radiotherapy often followed radical excision of the tumor. Markedly, ERMS of the larynx appears to be highly responsive to chemotherapy. However, further studies are required in order to improve the comprehending of their biological behaviors and to draft the optimum therapeutic approach. Nowadays, the experiment of anticancer drug sensitivity in vitro is the evidence in the chemotherapy and has become a highlight for the realization of individual tumor treatment [28, 29]. In our case, the tumor relapsed via microlaryngoscopic surgery combined with initial chemoradiotherapy. Therefore, we adopted more aggressive surgical intervention, and adjusted chemotherapy regimen according to the chemosensitivity assay in vitro (Fig. 2d). Finally, the patient has been remaining disease-free until now. Totally, the optimal therapy for laryngeal RMSs in adults is a multimodal approach comprising surgery followed by chemo- (in line with the anticancer drug sensitivity) and/or radiotherapy.

Although the survival rate of RMSs has been improved from 25% in 1970 to approximately 75% today, local tumor recurrence and metastasis remained challenging. Some authors noted that micro-metastasis is presumed leading to the high rate of failure with surgery alone [30]. Prognostic indicators, including age, tumor location, histologic subtype and response to treatment, have been identified. Hawkins [31] reported that patients with age < 20 years and those with a tumor size < 5 cm had a better prognosis. Multi-institutional pediatric trials have showed a 70% cure rate in children without metastatic disease, whereas adults had a poor prognosis. This may be implicated in the discrepancy of histologic variation that unfavorable subtypes (such as the pleomorphic RMSs) are more common in adults [32]. The embryonal subtype has a better overall prognosis associated with earlier age of onset, and the alveolar subtype, which accounts for the least, tends to carry the worst prognosis. Notably, laryngeal RMSs are supposedly less aggressive than RMSs elsewhere, presumably due to the cartilaginous borders of the larynx restricting local tumor spread [13]. Reviewing the 22 cases in the English-language literature, there were 21 cases receiving treatment (surgery, chemotherapy, radiotherapy or combination therapy) with 2 cases being lost to follow-up. Among these 19 cases, 17 cases were alive and disease-free at the ending of follow-up; 2 patients died of extensive distant metastases, and the pathological type was alveolar RMS and pleomorphic, respectively. The present case here did neither show systemic metastasis and is alive with disease-free after more than 18 months of follow up. However, late relapses warrant long-term follow up.

This was an unusual occurrence of an ERMS in the larynx of a middle-aged man who was a non-smoker. Misdiagnosis and missed diagnosis of laryngeal RMSs might appear when tumors presented as smooth protuberance. Therefore, it is important for otolaryngologists to remain vigilant and to suspect, confirm, and localize these tumors. Presently, multidisciplinary treatment based on the anticancer drug sensitivity remains the mainstay of the management, and offers best results to the patients. This is ideal, but not represent the reality around the word.

## Data Availability

All data generated or analysed during this study are included in this published article.

## Abbreviations

RMSs: Rhabdomyosarcomas
ERMS: Embryonal rhabdomyosarcoma
EMA: Smooth muscle actin
MRI: Magnetic resonance imaging
CT: Computed tomography
F: Female
M: Male
Gy: Gray
cGy: Centigray
YR: Year
HE: Hematoxylin-eosin staining
